# Wearable Sensor Data Classification for Identifying Missing Transmission Sequence Using Tree Learning

**DOI:** 10.3390/s23104924

**Published:** 2023-05-20

**Authors:** Kambatty Bojan Gurumoorthy, Arun Sekar Rajasekaran, Kaliraj Kalirajan, Samydurai Gopinath, Fadi Al-Turjman, Manjur Kolhar, Chadi Altrjman

**Affiliations:** 1 Department of Electronics and Communication Engineering, KPR Institute of Engineering and Technology, Coimbatore 641407, Tamilnadu, India; 2 Department of Electronics and Communication Engineering, Karpagam Institute of Technology, Coimbatore 641105, Tamilndu, India; 3 Artificial Intelligence Engineering Department, AI and Robotics Institute, Near East University, Mersin 10, Turkey; 4 Research Center for AI and IoT, Faculty of Engineering, University of Kyrenia, Mersin 10, Turkey; 5 Department Computer Science, College of Arts and Science, Prince Sattam Bin Abdulaziz University, Al Kharj 11990, Saudi Arabia; 6 Chemical Engineering Department, University of Waterloo, Waterloo, ON N2L 3G1, Canada

**Keywords:** classification learning, data accumulation, data sequence, transmission error, wearable sensors

## Abstract

Wearable Sensor (WS) data accumulation and transmission are vital in analyzing the health status of patients and elderly people remotely. Through specific time intervals, the continuous observation sequences provide a precise diagnosis result. This sequence is however interrupted due to abnormal events or sensor or communicating device failures or even overlapping sensing intervals. Therefore, considering the significance of continuous data gathering and transmission sequence for WS, this article introduces a Concerted Sensor Data Transmission Scheme (CSDTS). This scheme endorses aggregation and transmission that aims at generating continuous data sequences. The aggregation is performed considering the overlapping and non-overlapping intervals from the WS sensing process. Such concerted data aggregation generates fewer chances of missing data. In the transmission process, allocated first-come-first-serve-based sequential communication is pursued. In the transmission scheme, a pre-verification of continuous or discrete (missing) transmission sequences is performed using classification tree learning. In the learning process, the accumulation and transmission interval synchronization and sensor data density are matched for preventing pre-transmission losses. The discrete classified sequences are thwarted from the communication sequence and are transmitted post the alternate WS data accumulation. This transmission type prevents sensor data loss and reduces prolonged wait times.

## 1. Introduction

Wearable sensors are used as integrated analytical devices with mobile connectivity. Wearable sensors are mostly used for point-of-care devices which monitor the actual health condition of the users. Wearable sensor data are collected from the devices which provide appropriate information for further processes [1]. Wearable sensor data contain various details such as sleep patterns, heart rate (HR), blood pressure rate, walking steps, and physical activity ratio of the users. Wearable sensor data are gathered from the devices for health monitoring and diagnosis processes [2]. Wearable sensor data contain the actual health conditions and situations of patients. Wearable sensor data reduce both time and energy consumption levels in data monitoring systems [3]. Various methods and techniques are used for the wearable sensor data-gathering process. The data mining approach is widely used which identifies the exact content and details of data and provides optimal information for monitoring systems [4]. The data mining approach analyzes the data which are gathered from sensor devices. The analyzed data produce the necessary data for detection, prediction, and diagnosis processes [5].

Wearable sensor data are commonly used in healthcare monitoring and diagnosis systems. Data transmission is a process that transfers data from one device to another digital device. The data transmission technique is used for the healthcare diagnosis process [6]. Wearable sensor data are transmitted to disease prediction and diagnosis processes. The data transmission technique creates an effective communication service among the devices. A priority-based wearable sensor data transmission method is used for the patient’s diagnosis process [7,8]. A feature extraction approach is implemented in the method which extracts the important features and patterns for sensor data. The extracted data produce optimal information based on priorities for the diagnosis process [9]. The feature extraction approach minimizes the latency in data classification and identification processes. An automated disease prediction method is used for the diagnosis process. A dynamic and interoperable communication framework (DICF) is used for wearable data transmission in healthcare centers. DICF controls the data based on conditions and provides necessary information for decision-making, prediction, and diagnosis processes. DICF maximizes the overall accuracy in the patient’s diagnosis process which enhances the performance range of healthcare centers [10,11].

Wearable sensor data detection is a process that identifies the missing sensor data in healthcare analysis systems. Wearable sensor data contain appropriate data which are used for diagnosis and disease identification processes [12]. Machine learning (ML) algorithms are used for the detection process. The long short-term memory (LSTM)-based approach is used for the missing wearable sensor data detection processes. The LSTM approach identifies the missing sensor data using key values [13]. The key values contain the actual health condition and situation of patients which are stored in the database for further processes. LSTM achieves high accuracy in missing data detection that improves the efficiency range of the systems [14]. A real-time tracking approach is also used for the missing data detection process. A sensor code is implemented in the data file which contains the id and details of the data. Data tracking reduces the overall data missing ratio in healthcare centers [15]. Wearable sensor data analysis techniques are used for precise health analysis processes. An unsynchronized sensor data analytics (USDA) model is used in healthcare systems. USDA analyzes the appropriate datasets which are required for diagnosis and treatment processes in hospitals. The contributions of the article are listed as follows:

- Designing a concerted data transmission scheme through pre-verification for discrete sensor information identification. This process is designed for reducing the discreteness in data transmission regardless of the accumulation intervals.
- Designing a learning-based classification for discrete and continuous data sequence detection for preventing losses and diagnoses errors in WS observations. The identified errors are rectified in the consecutive sensing and transmission intervals such that maximum accumulation and sensor data are provided for healthcare record update.
- Performing a comparative analysis using defined metrics for verifying the proposed scheme’s efficiency with the other methods discussed in the related works section.

The organization of the article is as follows: Section 2 discusses the previous works related to the proposed scheme pursued by the proposed scheme description in Section 3. Section 4 presents the data-based analysis and is followed by the comparative analysis in Section 5. Finally, Section 6 winds up with the conclusion.

## 2. Related Works

Fan et al. [16] designed a wearable motion attitude detection method based on the Internet of Things (IoT). The proposed method uses the three-dimensional acceleration data which are provided by smart devices. The smart devices analyze the actual condition and situation of the users which reduces the latency in the computation process. The time domain algorithm is used here to detect the motion posture of patients which produces relevant data for the attitude detection process. Experimental results show that the proposed method achieves high accuracy in the detection process.

Luo et al. [17] proposed an activity-based person identification method using multimodal data. The wearable sensor data are gathered from the devices which provide optimal data for the identification process. The actual activities, postures, and gestures of persons are identified using multimodal data. A multivariate squeeze and excitation network (MESNet) is used here to classify the activity types of the person. The proposed method maximizes the overall accuracy in person identification which enhances the entire system.

Ni et al. [18] developed a deep multi-branch two-stage regression network (DMTRN) energy expenditure (EE) estimation. The proposed approach is mainly used to predict the accurate EE which produces necessary information for healthcare centers. The DMTRN extracts the important features from electrocardiogram (ECG) scans. The proposed approach reduces both time and energy consumption levels in the computation process. The proposed DMTRN approach improves the performance level of the systems.

Wang et al. [19] introduced a new sensor data augmentation method for human activity recognition (HAR). The actual goal of the method is to minimize the coverage of the sampling space. The constructive learning framework is implemented in the method which evaluates the datasets which are required for HAR. The constructive learning framework reduces the computational cost in the data augmentation process. The introduced method increases the accuracy of HAR which enhances the efficiency and feasibility range of the systems.

Lamooki et al. [20] developed a single wearable sensor-based data analytic framework for automated quantification. The developed framework is commonly used for ergonomic risk factor analysis and assessment processes. The proposed framework is an end-to-end framework that evaluates the severity of risks and issues. Both risks and important characteristics of risks are predicted by the framework. The developed framework enhances the overall feasibility and efficiency range of the applications.

Steurtewagen et al. [21] proposed a condition-based maintenance (CBM) using wireless sensor data. A machine learning (ML) algorithm is used in CBM that improves the accuracy of the prediction process. The actual aim of the method is to predict the diseases in the early stage which reduces the complexity of the diagnosis process. The ML algorithm is mainly used to combine the sensor data which provide appropriate data for the prediction process. The proposed CBM maximizes the effectiveness ratio of the systems.

Asare et al. [22] introduced longitudinal data analysis for digital biomarkers using a smartphone. The introduced analysis is mostly used in health monitoring systems which provide important data for the diagnosis process. The introduced analysis method identifies the exact depression and mood rating range of patients in healthcare centers. The exact difference between depressed and non-depressed data from the database reduces the latency in the prediction process.

Alsiddiky et al. [23] designed a type of designed-based data transmission using a selective decision model for healthcare applications. The selective decision model identifies and analyzes the data which are collected from smart devices. The proposed method provides a perfect queuing technique that reduces the energy consumption level in healthcare applications. The designed method achieves high accuracy in decision-making and diagnosis processes.

Alqarni et al. [24] proposed an error-loss data fusion (EDF) technique for the patient monitoring system. Wearable sensor data are collected from devices that provide appropriate datasets for monitoring systems. The EDF technique is mainly used to improve the accuracy range in the diagnosis process. The proposed method minimizes the difficulties in the identification and classification processes. When compared with other techniques, the proposed EDF technique maximizes the significance and efficiency range of monitoring systems.

Kerdjidj et al. [25] developed an efficient fall detection method using wearable sensors for healthcare systems. The main aim of the proposal is to detect the actual activities and conditions of patients. The detected data provide optimal information for activity classification and diagnosis process. A compressive sensing (CS) method is implemented here to minimize the energy-consuming range in the systems. The developed method achieves high accuracy in fall detection which enhances the performance ratio of the healthcare systems.

Sajedi et al. [26] presented a fuzzy-based data aggregation scheme (F-LEACH) for Internet of Things (IoT)-enabled healthcare systems. The wireless sensor network (WSN) is used in the IoT which provides a feasible services to patients. The WSN gathers the data which are presented in IoT healthcare systems. The developed method maximizes the lifetime level of the healthcare systems. Experimental results show that the proposed F-LEACH increases the reliability and feasibility range of the systems.

Shi et al. [27] introduced a gait recognition system, named FPRF-GR, using an inertial measurement unit (IMU). The introduced method identifies the exact walk, run, up, down, and stationary level of the person. The random forest (RF) algorithm is used here for the gait classification process. The RF algorithm reduces both time and energy consumption levels in the recognition process. The introduced FPRF-GR method increases the accuracy of the gait recognition process which improves the efficiency of the applications.

Shawen et al. [28] proposed a new Parkinson’s disease (PD) symptom detection method for healthcare systems. The actual goal of the proposed method identifies the exact PD symptoms which provide the necessary information for the early diagnosis process. Wearable sensors are used for PD detection which reduces the latency in identification and classification processes. The proposed method maximizes the effectiveness and feasibility level of healthcare systems.

Ross et al. [29] developed unsupervised multi-modal representation learning for healthcare systems. A two-stacked convolutional autoencoder is used here which identifies the important characteristics of the recognition process. The encoder classifies the actual types and classes of healthcare data. Moreover, the works in [8,30] suggests the continuous transmissions. The proposed model minimizes the latency ratio in the computation process. When compared with other models, the proposed model enhances the efficiency and feasibility level of the systems.

The methods discussed above focus on continuous transmissions and data accumulations for preventing discreteness in sequences. The discreteness is addressed through retransmissions and successive mode of sensor replacement in any validation. Therefore, the previous data correlation or error causing sequences in the identified intervals are to be mitigated for preventing precise health data augmentation. Though the sensing intervals and data are precise, the transmission failures result in inaccurate results. Therefore, the proposed scheme is designed to balance both discreteness and transmission failures for preventing sensor data accumulation flaws.

## 3. Concerted Sensor Data Transmission Scheme

### 3.1. Scheme Overview

**Figure 1:**
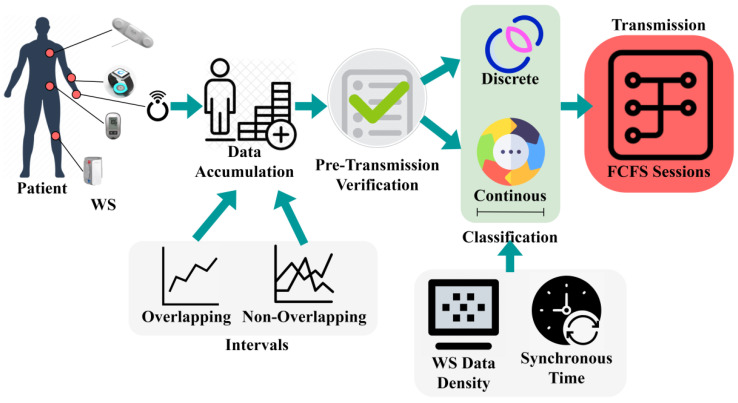
Proposed scheme illustration.

To determine the health status of patients and elder people, wearable sensors (WS) data accumulation and transmission are important. Wearable devices are paragoned to gather data on users’ health and daily routine of exercise and also even can send a patient’s health status to the doctor or healthcare in real-time. Wearable sensors technology allows the doctor or healthcare center to predict crucial health events and also predict diseases in their starting stages. This WS helps doctors or healthcare workers provide their patients with needed treatment on time which results in increasing the lifespan of the patients and ultimately protecting their life. Through particular time intervals, the continuous observation sequences provide a perfect diagnosis result. The sequence may get interrupted when there are abnormal events or sensors or communication failures of the devices or even overlapping of the sensing intervals. Therefore, by considering the importance of the continuous data collection and transmission sequence for WS, this article introduces a Concerted Sensor Data Transmission Scheme (CSDTS). Classification learning is a detective algorithm problem where the label of the class is forecasted for a particular input data. The way of organizing and gathering data into precise classification makes easier the process of placing and regaining the patients’ information, making it simple to use and protect. It is vital in predicting the correct information of the given patients’ data. In classification learning, the method is completely trained using the training data of the patient’s health, and then it is examined on the test data before being used to execute detection on new patients’ unseen health data. The data which are accumulated by the wearable sensor are ECG, heart rate of the patients, body temperature, oxygen red, blood pressure, and respiration rate. Figure 1 illustrates the proposed scheme with appropriate components and processes.

Wearable sensors are continuously more comfortable for patients to wear and less prominent for monitoring patients’ health or wellness without disturbing their daily life routine. The data which are collected from the patient through WS are transmitted through the wireless network to the healthcare center for analysis. This process forms the data accumulation process as presented in the above Figure. The data comprise both overlapping and non-overlapping information based on the sensor operation intervals. Such collected data accumulation provides fewer chances of missing data. The outcome of the data accumulation process helps in the pre-transmission verification process. In the transmission scheme, a pre-verification of accumulated data sequences is performed using the classifier algorithm. The pre-verification process observes the sequence whether it is discrete or continuous. By using the tree classifier algorithm, the missing data will be identified from the sequence. In the classification learning procedure, the aggregation and transmission interval coordination and sensor data density are matched for preventing pre-transmission losses. There it verifies whether the sensed data and the transferred data are the same while processing the pre-transmission verification procedures. From this output, the data transmission process takes place. The precise information of the patients is transferred to the healthcare center (Figure 1). In the transmission process, allocated first-come-first-serve-based sequential communication is pursued. The discrete classified sequences are frustrated from the communication sequence and are transmitted after the alternate WS data agglomeration.

### 3.2. Discussion of the Proposed Scheme

**Figure 2:**
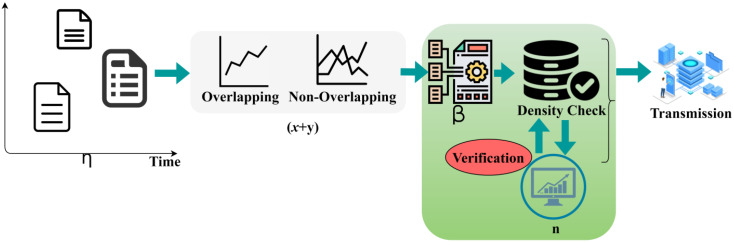
Pre-transmission verification process.

The wearable sensors determine the health status of the person who wears it and thus transmit the information of the health of the person to the healthcare without interrupting their daily life. From that, the data will be aggregated over the time intervals even when it is overlapping and non-overlapping. When the data are accumulated over the same time, then there happens to occur overlapping intervals. The data will be accumulated according to their health, and thus after transferring the data to the healthcare provider, they may take some steps to maintain the patient’s health. Then, this output will be helpful in the pre-transmission verification procedure to verify whether the data are accumulated precisely. This data accumulation plays a vital part in the data transmission procedure. The process of accumulating the data from the wearable sensors of the patients is explained by the following equations given below [9,24]:

(1) $$\left.\begin{array}{c} A^{i\varphi }=\alpha \left(\varphi \right)+i\beta \left(\varphi \right)\\ \left|\eta \right|=\sqrt{\alpha^{2}+\beta^{2}}\\ {\left|\eta \right|}^{2}=\eta \bar{\eta }=\alpha^{2}+\beta^{2} \end{array}\right\}$$

(2) $$\left.\begin{array}{c} \begin{array}{c} \gamma \left(\varphi \right)=\frac{x}{\alpha },\\ \gamma \left(\varphi \right)=\frac{y}{\alpha },\\ \gamma {\left(\varphi \right)}^{*}=\frac{y}{x} \end{array}\\ x=\alpha \gamma \left(\varphi \right)\\ y=\alpha \gamma {\left(\varphi \right)}^{*} \end{array}\right\}$$

where A is denoted as the sensors embedded over the patients, $\left|\varphi \right|$ is represented as the health status of the patients who wears the wearable sensors, η is denoted as the data accumulated from the patients, α is denoted as the time intervals when the data is accumulated, β is represented as the aggregation of the health status without interrupts, x is represented as the overlapping intervals, y is denoted as the non-overlapping intervals, and i is denoted as the operation done to accumulate the data from the patients without disrobing them. Now, this data accumulation based on the overlapping and the non-overlapping intervals output will be used in the pre-transmission verification process. In this process, it checks whether the sensed data and the transfer data are completely accumulated or missing. The data aggregation may engender lesser chances of missing data. It checks the sequence of the data from the patients to maintain their health. This process can be done by using the tree classifier algorithm to identify whether the sequence is continuous or discrete. If the data are collected on time and within the given limit, then it will not lead to missing data or discretion in the sequence of data. The pre-transmission verification process is illustrated in Figure 2.

**Figure 3:**
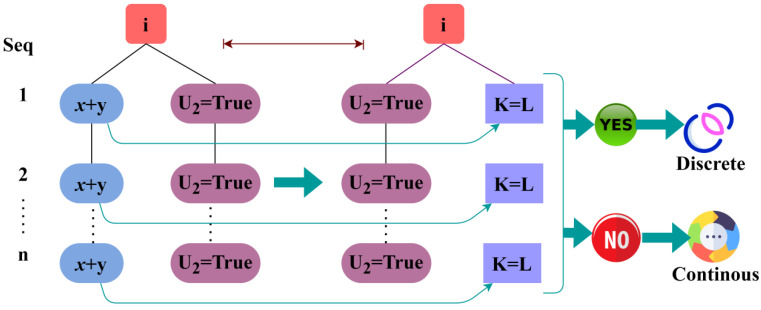
Classification of discrete and continuous sequences.

The pre-transmission verification procedure helps in finding the sequence of the data, and it determines whether the acquired data are perfect or not. Here, it verifies whether the data which are collected from the patients are correct and whether the data belong to them or not. From this, the transmission process proceeds further steps to enhance the patient’s health. The output of the pre-transmission verification procedure helps in the transmission of the health data of the patients to the healthcare center without any interruptions in the middle of the process (Figure 2). The accumulated data are considered for the transmission procedure which identifies the missing data in the data sequence data. For this process, tree classifier learning is used for regaining the data from the patients without any waiting time. This learning process is also used in reducing the aggregation error of the data from the patients. The procedure helps in determining the sequence of the data with the help of the classifier algorithm from the sensed data and the synchronous time of the health data of the patients. The process of pre-transmission verification based on the data accumulation is explained by the following equations given below:

(3) $$\left.\begin{array}{c} U_{1}U_{2}=(\alpha +i\beta )(\gamma +iZ)\\ =\left(\alpha Z-\beta \alpha \right)+i\left(\alpha \beta +\beta Z\right)\\ U_{1}+U_{2}=\left(\alpha +i\beta \right)\left(\gamma +iZ\right)\\ =\left(\alpha +\gamma \right)+i\left(\beta +Z\right)\\ U_{1}U_{2}=\left(g_{1}\alpha^{i\varphi_{1}}\right)\left(g_{2}\alpha^{i\varphi Z_{2}}\right)\\ =\left(g_{1}g_{2}\alpha^{i\varphi_{1}}+\alpha_{Z}\right)\\ U_{1}+U_{2}=\left(\left(g_{1}\alpha^{i\varphi_{1}}\right)+\left(g_{2}\alpha^{i\varphi Z_{2}}\right)\right)\\ =g_{1}\alpha \left(\varphi_{1}\right)+g_{2}\beta \left(\varphi_{2}\right)\\ =i(g_{2}\beta \left(\varphi_{2}\right)+g_{1}\alpha \left(\varphi_{1}\right) \end{array}\right\}$$

where Z is denoted as the pre-transmission verification procedure, $U_{1}$ is denoted as the output of the data accumulation process, $U_{2}$ is denoted as the checking of the sensed data, and g is represented as the status of the sensed data whether is perfect or not.

(4) $$\left.\begin{array}{c} \begin{array}{c} T_{\left(\alpha +i\beta \right)}=\left[\begin{array}{cc} \alpha & -\beta \\ \beta & \alpha \end{array}\right]\\ such\;that,\\ T\left(0+iZ\right)=\left[\begin{array}{cc} 0 & -1\\ 1 & 0 \end{array}\right] \end{array}\\ T\left(1+iZ\right)=\left[\begin{array}{cc} 1 & 0\\ 0 & 1 \end{array}\right]\\ \begin{array}{c} A_{\left(\alpha +i\beta \right)}T_{\left(\gamma +iZ\right)}=\left[\begin{array}{cc} \alpha & -\beta \\ \beta & \alpha \end{array}\right]\left[\begin{array}{cc} \gamma & -Z\\ Z & \gamma \end{array}\right]\\ =\left[\begin{array}{cc} \alpha \gamma -\beta Z & \beta \gamma +Z\gamma \\ \beta \gamma +Z\gamma & \alpha \gamma -\beta Z \end{array}\right] \end{array} \end{array}\right\}$$

where T is denoted as the outcome of the pre-transmission verification procedure. Now by considering the WS data density and the synchronous time of the data, the sequence of the accumulated data can be found. In the health data transmission scheme, a pre-verification of continuous or missing transmission sequences is identified using a classification tree learning algorithm. In this learning process, the accumulation and relaying interval simultaneousness and sensed data density are equivalent to precluding the losses in the pre-transmission verification procedure. The WS data density procedure is used to verify whether the accumulated data and the transfer data are the same. They should not be different, which can cause the error in the procedure. Here, the density is represented as the amount of data accumulated from the patients and its values for an idiosyncrasy. Figure 3 presents the classification for discrete and continuous sequences.

**Figure 4:**
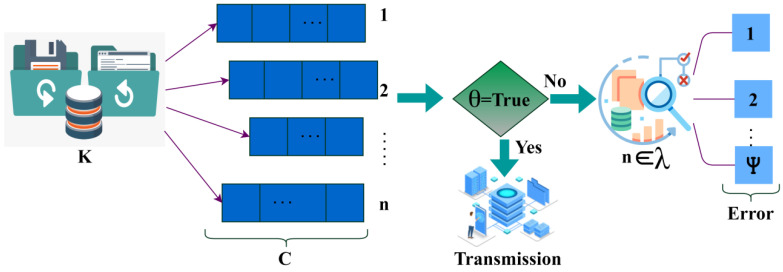
Error detection in continuous sequence.

The WS data density validation checks the percentage of the acquired data and the sequence of the patient’s data aggregation. The verification of whether the sensed data and the processed transfer data are the same or not will be useful in the determination of the sequence of the data. After identifying the WS data density, the output can be used in the process conducted by the classifier learning technique. Then, the data transmission procedure takes place along with the time taken for the data accumulation. This data density identification helps in determining the missing data in the sequence (Figure 3). The process of WS data density observation for the identification of the data sequence is explained by the following equations given below:

(5) $$\left.\begin{array}{c} \begin{array}{c} K:Y\to Z\\ L\left(K\right)=\overset{\alpha_{i}}{\underset{T}{\int }}L\left(T\right)\alpha^{i\alpha \beta }dT\\ L\left(K\right)=\underset{T=0}{\sum }L\left(T\right)\alpha^{i\beta }Ky \end{array}\\ Z\left(\alpha \right)=\underset{T=0}{\sum }L\left(T\right)\alpha^{-t}\\ \gamma \left(T\right)=\underset{T=0}{\sum }L\left(T\right)\beta \left(\frac{\varphi K\left(2T+1\right)}{2Z}\right) \end{array}\right\}$$

where K is denoted as the identification of the WS data density and L is denoted as the sensed data.

(6) $$\left.\begin{array}{c} W_{j}=\varepsilon \left(W_{j}\right)\\ =V\left(W\right)\\ \begin{array}{c} =\left[0\ldots 0\;1\;0\ldots 0\right]\\ =\left[\begin{array}{ccc} W_{11} & \ldots & W_{1Z}\\ W_{21} & \ldots & W_{2Z}\\ \begin{array}{c} \vdots \\ W_{nj} \end{array} & \begin{array}{c} \ddots \\ \ldots \end{array} & \begin{array}{c} \vdots \\ W_{nZ} \end{array} \end{array}\right] \end{array} \end{array}\right\}$$

where W is denoted as the data which is going to be transferred to the healthcare center, ε is denoted as the comparison of the sensed data and the transfer data, V is denoted as the output of the WS data density identification process, n is represented as the percentage of the accumulated data, and j is denoted as the percentage of the acquired data. Now along with the WS data density identification, the synchronous time of the data will be identified. This time is represented as the different types of sensors producing the data at the same time. The patients’ wearable sensors provide different data at the same time along with the WS data density detection procedures. The time taken for the identification process is less by using the classifier learning algorithm, and thus it helps in the reduction of the aggregation error. The outcome of the WS data density and the synchronous time detection will be helpful in the determination of the data sequence to transmit the patient’s health data to the healthcare center. The process of determining the synchronous time of the accumulated data to identify the sequence of the data is explained by the following equations given below:

(7) $$\left.\begin{array}{c} U_{t}=\theta \left(W_{u}+U_{t}+\beta_{t}\right)\\ i_{T}=\theta \left(W_{i}+U_{Z}+\beta_{i}\right)\\ \begin{array}{c} \theta_{t}=\left(W_{V}+V_{0}+\beta_{0}\right)\\ \gamma_{T}=F_{Z}U_{1-0}+i_{T}\left(W_{u}+U_{t}+\beta_{t}\right)\\ \begin{array}{c} \gamma_{Z}=\theta_{t}\left(W_{j}\right)\\ L_{T}=\left(x_{1}W_{Z}+U_{Z}+\beta_{Z}\right)\\ \begin{array}{c} g_{T}=\theta_{t}\left(W_{g}+U_{g}+\beta_{g}\right)\\ \gamma_{T-Z}=Z_{T}\cdot g_{T-1} \end{array} \end{array} \end{array} \end{array}\right\}$$

(8) $$\left.\begin{array}{c} \begin{array}{c} \begin{array}{c} E*g=\overset{1}{\underset{0}{\int }}L\left(T\right)g\left(T-\theta \right)dT\\ X\left[\eta \right]*Z\left[\eta \right]=\underset{j=0}{\sum }X\left[j\right]\cdot T\left[\eta -j\right]\\ =\overset{i}{\underset{j=0}{\sum }}Z\left[j\right]\cdot X\left[\eta -j\right] \end{array}\\ \phi =\eta \left(X*Z_{ij}\right)\\ \underset{i=0}{\sum }\left(Z\right)=\underset{j=0}{\sum }\left(Z_{j}\ldots Z_{\alpha }\right) \end{array}\\ \underset{ij}{\sum }\left(Z\right)=\frac{1}{T}\underset{j=1}{\sum }Z_{j} \end{array}\right\}$$

where θ is denoted as the identification of the synchronous time of the accumulated data, E is denoted as the outcome of the synchronous time detection procedure, and F is represented as the different sensors. Now based on both the WS data density verification process and the synchronous time detection procedure, the tree classifier learning technique determines the sequence of the data, whether the sequence of the data is discrete or continuous. Figure 4 presents the error detection in the continuous sequence.

**Figure 5:**
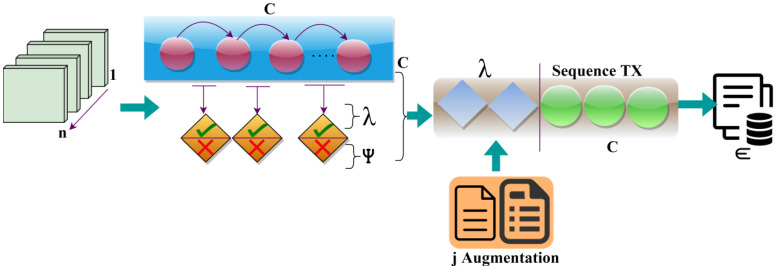
Data transmission from  n process.

The learning process helps in detecting the missing data if the data happen in sequence. Then, a further process is done to reduce the missing data occurrence and errors in the procedure. If there are any mistakes in the process happening, then the data can be missed from the wearable sensors. The detection of the missing data before the transmission process is conducted efficaciously by the learning method, and then the errors are identified that cause the missing data (Figure 4). The output of the classification process will be helpful in the transmission process where the data of the patients are transmitted to the healthcare center. The process of determining the discrete data by the learning technique is explained by the following equations given below:

(9) $$\left.\begin{array}{c} \begin{array}{c} \lambda \left(Z^{\prime },Q^{\prime }\right)=\left(L,\left(Z^{\prime },Q^{\prime }\right)\right)\\ L,\left(Z^{\prime },Q^{\prime }\right)=\frac{Q^{\prime }L^{\prime }T}{\sqrt{\beta }}\\ \mathrm{where}, \end{array}\\ \lambda =\left[Z_{1}\ldots Z_{T}\right]\\ \overset{\lambda }{\underset{i=1}{\sum }}|Z_{i}{|}^{2}=1 \end{array}\right\}$$

(10) $$\left.\begin{array}{c} \psi_{j}\left(T\right)=\frac{|T_{i}{|}^{2}}{1+\sum_{x=1}|T_{\alpha }{|}^{2}}\\ =\frac{L{\left(Z_{j}\right)}^{2}+T{\left(Z_{j}\right)}^{2}}{1+\sum_{i=1}{\left(L_{\alpha }\right)}^{2}+{\left(Z_{\alpha }\right)}^{2}}\\ \begin{array}{c} \psi \left(Z\right)=\frac{{\left|Z\right|}^{2}}{1+{\left|\left|Z\right|\right|}^{2}}\\ =\frac{L\left(Z\right)+T\left(Z\right)}{1+L\left(Z\right)+\left(Z\right)} \end{array} \end{array}\right\}$$

where λ is denoted as the identification of the discrete data sequence and ψ is denoted as the error that happened during the procedure. Now, the continuous sequence of the data can be determined based on the WS data density and the synchronous time detection procedures. If there are no errors in the process, then there will be no missing data in the sequence, and the sequence will be continuous. Based on the outcome of the data accumulation from the patients who wear the sensors based on the overlapping and the non-overlapping intervals, the pre-transmission verification process takes place. By using the tree classifier algorithm, the data sequence can be determined whether any of the data are missing or whether all the data are acquired precisely. Depending on the sequence of the data, the data transmission process takes place. The process of identifying the continuous data sequence for the data transmission process is explained by the following equations given below:

(11) $$\left.\begin{array}{c} C\left(K,z\right)=\frac{<K∕Z>}{\sqrt{\beta }}\\ =\frac{K\cdot \bar{Q}}{\sqrt{\beta }}\\ =\overset{\beta }{\underset{i=1}{\sum }}K_{j}\bar{Q_{j}}∕\sqrt{\beta } \end{array}\right\}$$

(12) $$\left.\begin{array}{c} \mu \left(L\right)=Z\\ \mu \left(Z\right)=\gamma \left(Z\right)=\frac{\alpha \left(Z\right)}{\beta \left(Z\right)}\\ \begin{array}{c} \mu \left(Z\right)=LU\left(Z\right)\\ =L\left(T\left(Z\right)+L\left(Z\right)\right)i\\ =\left[\underset{ij}{\sum }\left(0,T\left(Z\right)\right)i+\underset{ij}{\sum }\left(0,L\left(Z\right)\right)j\right] \end{array} \end{array}\right\}$$

where C is denoted as the continuous sequence of the accumulated data and μ is represented as the outcome of the status of the data sequence. Based on the outcome of the pre-transmission verification process, the data transmission process takes place. The first-come-first-serve session is used in the data transmission procedure. Here, it transfers the health data of the patients to the healthcare center. From the accumulated data, the healthcare center finds the status of the health of the patient and determines the way to enhance their health. The discrete classified sequences are balked from the sequence of communication and are transmitted after the alternate WS data aggregation. The process of data transmission to the healthcare center from the wearable sensors is explained by the following equations given below:

(13) $$\left.\begin{array}{c} \in \left(Z\right)={\left|T\right|}^{2}\\ =L{\left(Z\right)}^{2}+T{\left(Z\right)}^{2}\\ \begin{array}{c} \in \left(Z\right)=\left|Z\right|=\sqrt{T{\left(Z\right)}^{2}+L{\left(Z\right)}^{2}}\\ \in \left(Z\right)=\mu \;U\left|Z\right|+\beta \frac{Z}{\left|T\right|}\\ \varepsilon \left(Z\right)=\gamma \left(\frac{\left|Z\right|}{T}\right)ij \end{array} \end{array}\right\}$$

(14) $$\frac{\partial C_{\theta }}{\partial \theta_{ij}}=\frac{\partial Q_{\vartheta }}{\partial \vartheta_{Q}^{K}}\frac{\partial \theta_{j}^{Q}}{\partial \mu_{ij}^{K}}$$

where ∈ is denoted as the data transmission process. This process helps in determining the health status of the patients through wearable sensors. The aggregation error and the wait time are reduced by using the tree classifier algorithm. The data transmission using the different sequences is presented in Figure 5.

The $\left(1\;to\;n\right)$ sequence is classified for $\left(C,\;\lambda ,\;\psi \right)$ over the varying aggregation instances. The aggregation sequences are classified for C∈ first-come-first-serve transmission, and λ is induced for j augmentation. The augmented data is validated for its density before transmission slot allocation (Refer to Figure 5). The data accumulated from the patients helps in the transmission process by identifying the sequence data. The missing data will be identified, and thus further steps are taken to enhance the process without any errors. These processes take place by using the tree classifier algorithm to reduce the sensor loss with the data aggregation loss. This also reduces the wait times in the process of data transmission to healthcare.

## 4. Data Analysis

The proposed scheme is analyzed using the dataset [31] that gives information on patient activity monitoring. The logs are generated using 10 min observation intervals of different aged people. For a maximum of eight day-to-day activities, the sensor information is used for varying the activities as precise/abnormal. The missing sequences are identified across various sequences (28 in this analysis) for a maximum of 60 min time. In Table 1, the actual sequence required for the activity identification and acceptable λ.

**Figure 6:**
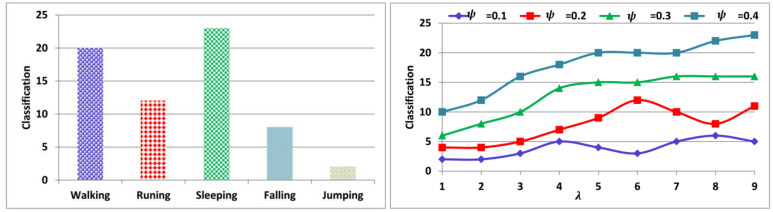
Classification for activities and  λ.

The actual sequence required and the discrete sequences observed are tabulated in Table 1. The possible errors are also presented in the above for which the activity recognition is presented. Depending on W and θ verification, the λ and $\psi \notin \left(x+y\right)$ are identified. If λ is less than  ψ then the sequence cannot be used for predicting the activity. The red-highlighted entries indicate the error occurred sequences. Therefore, the pre-transmission verification is performed for the different activities as presented in Figure 6.

**Figure 7:**
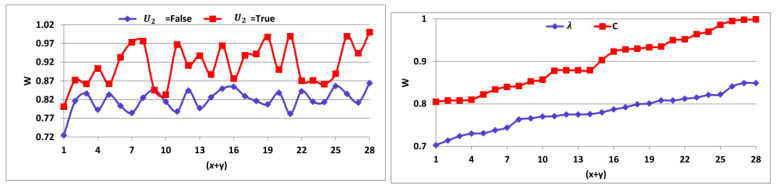
W matching analyses.

The above classification is performed based on the activity and λ for which ψ is suppressible. Considering W and β, the F-based accumulations and j augmentations are induced. Therefore, ψ increases the chances of classification and hence the activity requirements. The variable m is the activity which features more classification for various $U_{1}$ and $\;U_{2}=true$ instances (Refer to Figure 6). Now, the $\left(x+y\right)$ combination’s impact over  W matching analysis is presented in Figure 7.

The W matching is high for C and $U_{2}=True$ sequences compared to $U_{2}=$ false and λ validations. The $U_{2}=$ false and λ variables are required for classifications preventing errors. Therefore, multiple instances of verification are required for improving the data augmentation and reducing losses (Figure 7).

## 5. Results and Discussion

In this section, metric-based comparative analysis is presented for validating the proposed scheme’s performance. The metrics of data accumulation, classifications, data loss, transmission wait time, and discrete sequence detection are used for this analysis. The intervals are varied up to 60 min, and the sequences are varied up to 28 including overlapping and non-overlapping. The alongside considerations are EDF [25], F-LEACH [27], and SDAM [19] from the related works section.

### 5.1. Data Accumulation

**Figure 8:**
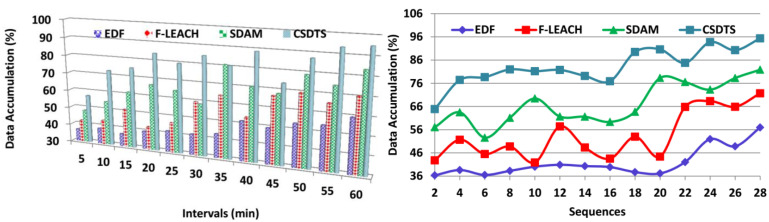
Data accumulation comparisons.

The accumulation of the data is efficacious from the patients through the wearable sensors for the data transmission process. The data are accumulated based on the overlapping and the non-overlapping intervals, and thus it will be considered the pre-transmission verification process. When the data are accumulated over the same time, then there happens there is an overlapping interval. The data will be accumulated according to their health, and thus after transferring it to the healthcare provider then they may take some steps to maintain the patient’s health. From this, the treatment will be given to the patients if there are any issues according to their health. This helps in the increment of the lifespan of the patients. Then, this output will be helpful in the pre-transmission verification procedure to verify whether the data are accumulated precisely. Based on the data accumulation process, the classification process also takes place; there it determines the sequence of the data. From the outcome of the classification process, the data transmission process is conducted by using the learning technique (Figure 8).

### 5.2. Classifications

**Figure 9:**
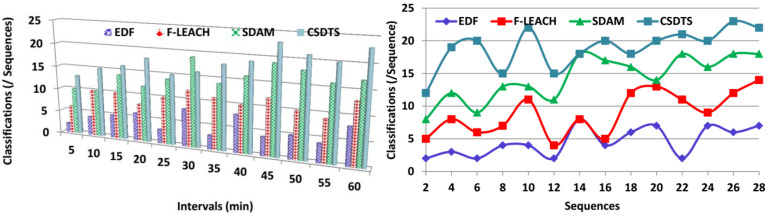
Classification comparisons.

The classification process is efficacious in this method by using the tree classifier algorithm to identify the discrete and the continuous sequence of data. Based on the WS data density and the synchronous time, the classification process takes place. The WS data density is the checking process whether sensed data and the transfer data are the same. The synchronous data time is the one where the different sensors produce the data at the same time. Depending on the classification process, the data transmission process is taken where the health status of the patients is sent to the healthcare center. In the health data transmission scheme, a pre-verification of continuous or missing transmission sequences is identified using a classification tree learning algorithm. In this learning process, the accumulation and relaying interval simultaneousness and sensed data density are equivalent to precluding the losses in the pre-transmission verification procedure. In this way, the classifications are better in this process for the data transmission procedure (Figure 9).

### 5.3. Data Loss

The loss of data is lesser in this process by using the learning technique with precise information about the patient’s health. After accumulating the data from the patients based on the overlapping and the non-overlapping intervals, the data will be checked whether they are perfect or not. So, by checking the data, the loss of the data can be reduced.

**Figure 10:**
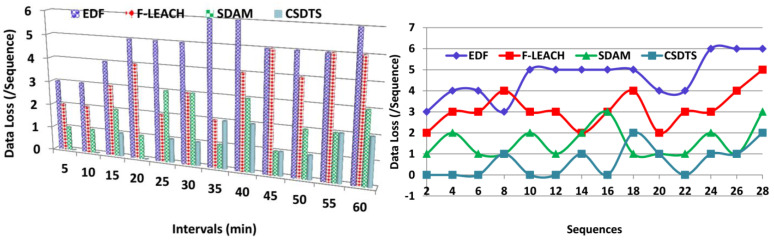
Data loss comparisons.

The intervals when the data are accumulated are maintained without leading to the overlapping of the time. Based on this output, the pre-transmission and classification process takes place. If there are any mistakes in the process happening, then the data can be missed from the wearable sensors. The detection of the missing data before the transmission process is done efficaciously by the learning method, and then the errors are identified that cause the missing data. Thus, that will be helpful in the reduction of data loss. The classification learning technique helps in the determination of the missing data and takes some steps to enhance the process to reduce the missing data and identify it (Figure 10).

### 5.4. Transmission Wait Time

**Figure 11:**
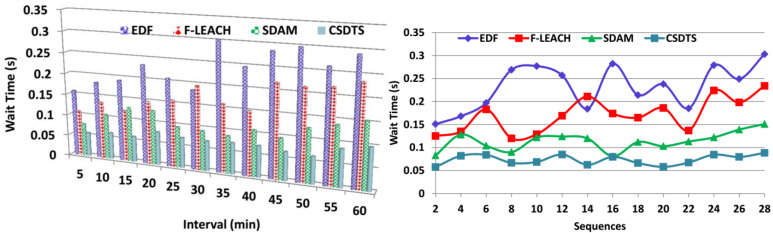
Transmission wait time comparisons.

The wait time is less in this process by using the learning algorithm. The output of the classification process will be helpful in the transmission process where the data of the patients are transmitted to the healthcare center. The data aggregation may engender lesser chances of missing data. It checks the sequence of the data from the patients to maintain their health. This process can be done by using the tree classifier algorithm to identify whether the sequence is continuous or discrete. Depending on the outcome of the pre-transmission and the classification process, the data transmission process takes place. In this process, the first-come-first-serve method is pursued. From the accumulated data, the healthcare center finds the status of the health of the patient and determines the way to enhance their health. The discrete classified sequences are balked from the sequence of communication and are transmitted after the alternate WS data aggregation. By enhancing the process by using the learning algorithm, the wait time for the data transmission is made less (Figure 11).

### 5.5. Discrete Sequences Detection

**Figure 12:**
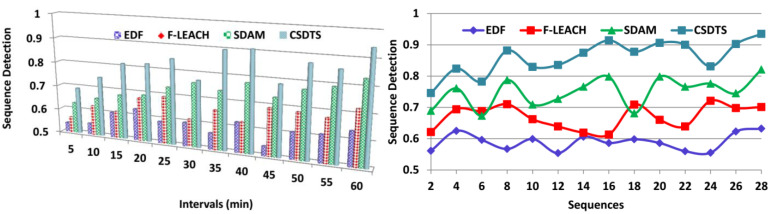
Discrete sequence detection comparisons.

The missing data in the sequence identification are better in this method with the help of the tree classifier algorithm technique. The learning process helps in detecting the missing data if it happens in the sequence. The detection of the missing data before the transmission process is conducted efficaciously by the learning method, and then the errors are identified that cause the missing data. Based on the data accumulation output, the pre-transmission verification process takes place where the discrete and the continuous data sequence is determined. Here, it verifies whether the data which are collected from the patients are correct and whether the data belong to them or not. From this, the transmission process proceeds further steps to enhance the patient’s health. It also checks whether the data which are accumulated have the complete information of the patients who wear the wearable sensors. The discrete sequence can be identified based on the WS data density detection and the synchronous time detection. So, after these processes, the data transmission procedure takes place with the first-come-first-serve method (Figure 12).

Analysis Summary: The proposed scheme improves data accumulation, classification, and sequence detection by 11.93%, 14.98%, and 11.12%, respectively. This scheme reduces data loss and waits time by 9.52% and 9.42%, respectively.

## 6. Conclusions

This article projected a concerted sensor data transmission scheme for wearable sensor-based applications. The proposed scheme focused on health data aggregation and transmission by thwarting the loss impact of discrete observation sequences. For improving the medical diagnosis based on sensed data, this scheme performs data aggregation from overlapping and non-overlapping intervals for reducing data loss. Therefore, a pre-transmission verification using classification learning is performed for identifying the sequences. The continuous sequences are allocated with a first-come-first-serve transmission interval. Contrarily, the discrete sequence is augmented with further sensed data for preventing data loss. This process is performed after the classification for preventing losses and transmission wait times. The transmission data are validated using their accumulated density and synchronization interval for preventing losses. Such discrete error-causing sequences are mitigated from the first transmission, and data augmentation is performed. Therefore, the proposed scheme improves data accumulation, classification, and sequence detection by 11.93%, 14.98%, and 11.12%, respectively. This scheme reduces data loss and waits time by 9.52% and 9.42%, respectively.

## Figures and Tables

**Table 1:** Sequence required and λ.

**Activity**	**10 min**			20 min			30 min			40 min			50 min			60 min		
	*n*	*λ*	*ψ*	*n*	*λ*	*ψ*	*n*	*λ*	*ψ*	*n*	*λ*	*ψ*	*n*	*λ*	*ψ*	*n*	*λ*	*ψ*
Walking	26	12	3	27	12	5	29	17	4	32	6	1	39	10	3	53	4	1
Running	34	9	2	39	13	0	48	6	3	52	9	5	55	5	1	64	11	13
Sleeping	17	2	0	20	0	0	26	3	0	26	0	0	28	0	0	33	1	0
Falling	29	4	1	36	6	3	48	9	14	59	5	7	64	7	1	67	6	2
Jumping	27	2	4	32	7	2	32	14	7	37	17	3	48	8	0	57	2	0

## Data Availability

Not applicable.
